# Predictors of delayed disclosure of rape in female adolescents and young adults

**DOI:** 10.3402/ejpt.v6.25883

**Published:** 2015-05-11

**Authors:** Iva A. E. Bicanic, Lieve M. Hehenkamp, Elise M. van de Putte, Arjen J. van Wijk, Ad de Jongh

**Affiliations:** 1 National Psychotraumacenter for Children and Youth, University Medical Center Utrecht, Utrecht The Netherlands; 2 Department of Paediatrics, University Medical Center Utrecht, Utrecht, The Netherlands; 3 Department of Behavioral Sciences, ACTA, University of Amsterdam and VU University, Amsterdam, The Netherlands; 4 School of Health Sciences, Salford University, Manchester, United Kingdom

**Keywords:** Adolescents, young adults, rape, sexual assault, disclosure, latency to disclosure, posttraumatic stress disorder

## Abstract

**Background:**

Delayed disclosure of rape has been associated with impaired mental health; it is, therefore, important to understand which factors are associated with disclosure latency. The purpose of this study was to compare various demographics, post-rape characteristics, and psychological functioning of early and delayed disclosers (i.e., more than 1-week post-rape) among rape victims, and to determine predictors for delayed disclosure.

**Methods:**

Data were collected using a structured interview and validated questionnaires in a sample of 323 help-seeking female adolescents and young adults (12–25 years), who were victimized by rape, but had no reported prior chronic child sexual abuse.

**Results:**

In 59% of the cases, disclosure occurred within 1 week. Delayed disclosers were less likely to use medical services and to report to the police than early disclosers. No significant differences were found between delayed and early disclosers in psychological functioning and time to seek professional help. The combination of age category 12–17 years [odds ratio (OR) 2.05, confidence intervals (CI) 1.13–3.73], penetration (OR 2.36, CI 1.25–4.46), and closeness to assailant (OR 2.64, CI 1.52–4.60) contributed significantly to the prediction of delayed disclosure.

**Conclusion:**

The results point to the need of targeted interventions that specifically encourage rape victims to disclose early, thereby increasing options for access to health and police services.

Previous studies have shown that disclosure of rape to formal agencies, such as police or mental health services, is uncommon (Fisher, Cullen, & Turner, 2000; Wolitzky-Taylor et al., 2011), especially when the rape has been committed on a date or by an acquaintance and involves the victim's use of drugs and/or alcohol (Resnick et al., 2000; Wolitzky-Taylor et al., 2011). There is evidence to suggest that victims believe that professionals will not be helpful to them because their rape experience does not match stereotypical conceptions of rape, such as involving a stranger, a weapon, and severe injury (Patterson, Greeson, & Campbell, 2009; Resnick et al., 2000). Accordingly, adolescents and young adults, who are more at risk to be victimized by rape than other age groups (De Haas, Van Berlo, Bakker, & Vanwesenbeeck, 2012; Tjaden & Thoennes, 2006), may not receive targeted mental health care and may not report the crime to the police (Ruch, Coyne, & Perrone, 2000).

For reasons of mental health and public safety, it is important to understand the potential factors that are related to disclosure. Timing of disclosure may be a crucial factor, as early disclosers are more likely to utilize appropriate medical care and report to the police than delayed disclosers (Ahrens, Stansell, & Jennings, 2010; Ullman & Filipas, 2001). In contrast, adults who wait longer than 1 month to disclose the rape are more likely to suffer from posttraumatic stress disorder (PTSD) and depression compared to early disclosers (Ruggiero et al., 2004). In addition, adolescents who disclose their rape experience at least 1 month after the incident took place are found to be at higher risk for major depressive disorder and delinquency (Broman-Fulks et al., 2007) compared to those who disclosed within 1 month.

Victim–assailant relationship is crucial in disclosure latency, with victims being at higher risk for delayed disclosure if there is a close relation with the assailant (Kogan, 2004; Koss, 1988; Rickert, Wiemann, & Vaughan, 2005). In contrast, delayed disclosure is less common in victims of a stereotypical rape, i.e., rape by a stranger including a weapon and injury (Smith et al., 2000). Victims of prior sexual trauma are more likely to postpone disclosure of a subsequent assault than those without prior victimization (Smith et al., 2000; Ullman, 1996). This is in contrast with the findings of Ahrens et al. (2010), who report no difference in rates of prior sexual trauma between early and delayed disclosers. In addition, the victim's age appears to be an important variable in predicting disclosure. Evidence suggests that young children are at higher risk for delayed disclosure than adolescents (Kogan, 2004; Schönbucher, Maier, Mohler-Kuo, Schnyder, & Landolt, 2012). Thus, various rape and victim-related characteristics have been found to be associated with timing of disclosure.

The majority of the aforementioned studies included college and adult female rape victims. It is important to examine rape disclosure latency in an age and sex group that is most at risk for rape victimization. There is only one prior quantitative study in adolescents (those aged 12–17 years) that identified factors that might influence disclosure latency (Kogan, 2004). He found that identity of the assailant, a familial relationship with the assailant, and a history of drug abuse in the household were related to the timing of disclosure. The results suggested that a familial relationship with the assailant will postpone disclosure, whereas a history of drug abuse in the household, albeit this seems counterintuitive, makes prompt disclosure more likely. This study had some limitations, including the fact that the interviews were conducted by telephone and that the description of the relationship with the assailant was limited. Therefore, in the present study, we investigated a sample of female adolescent and young adult victims of rape who were admitted to a specialized mental health centre for victims of sexual assault. The first aim of this study was to compare demographics, post-rape characteristics, and psychological functioning between early and delayed disclosers in this group. The second aim, based on the exploratory findings of Kogan (2004), was to determine the predictors for delayed disclosure in adolescents and young adults, including age, prior trauma, and victim–assailant relationship using logistic regression analyses. Insight into the predictors for delayed disclosure for adolescents and young adults may reveal not only potential causal mechanisms but also possible targets for interventions that increase victims’ opportunities to receive timely post-rape services.

## Methods

### Subjects and data collection

Rape was defined as “an event that occurred without the victim's consent that involved the use or threat of force in vaginal, anal, or oral intercourse” (Tjaden & Thoennes, 2006). The definition includes both attempted and completed rape; the term “completed” referring to vaginal, oral, anal, or multiple penetrations. Victims who disclosed within 1 week were defined as “early disclosers,” whereas those who disclosed at least after 1 week were defined as “delayed disclosers.” This dichotomization of the variable “disclosure latency” was based on the study of Ahrens et al. (2010) and the national standard criteria for admission to a Rape Centre in the Netherlands, i.e., a maximum of 7 days post-rape.

The study was conducted in the Dutch National Psychotrauma Centre, which provides psychological services for rape victims aged 12–25 years and their parents. Between May 2005 and December 2011, the centre received 621 phone calls concerning alleged rape victims from police authorities, mental health services, and self-referrals. In 178 cases, the phone call did not result in admission at the centre because of age limitations, or motivational reasons. In 108 cases, referrals were made to other institutions because the index trauma was chronic childhood sexual abuse rather than rape in adolescence/young adulthood. Of the 335 cases admitted to the centre, 12 were not included in this study because of male gender, resulting in a final sample of 323 females with the index trauma being single rape. Referral sources for this final sample included the police (33.7%), mental health services (40.7%), and self-referrals, i.e., victims or parents (25.6%).

### Procedure

During admission, all patients underwent a psychological assessment, consisting of 1) a structured interview for obtaining demographic and post-rape characteristics and 2) self-report questionnaires to obtain information about mental health functioning. Information from the interview was transcribed onto a form designed for this purpose. The following variables were obtained and dichotomized or categorized for the purpose of the study:

#### Demographic and victim characteristics

We asked patients about their current age, educational level (lower, middle, or higher), and whether they were of Dutch origin (i.e., in case of having parents born in the Netherlands). Those between 12 and 17 years of age were defined as adolescents and those between 18 and 25 years of age as young adults. We also asked whether the patient was living with their parent(s) (yes/no), and whether the family structure was complete, i.e., whether the biological parents were living together (yes/no). Patients were then asked to confirm the presence of prior negative sexual experiences (yes/no), and whether they had a current sexual relationship (yes/no).

#### Rape characteristics

Information about date and time of the rape was obtained to calculate the time since rape at admission. Next, patients were requested to describe the rape. Their response was categorized into use of penetration (yes/no), group rape (yes/no), use of physical violence (yes/no), and use of threats verbally and/or with a weapon (yes/no). Also, information regarding the victim's relationship to the assailant was obtained. The assailant was defined as a stranger when the victim had never been in contact with the assailant before the rape. Responses were used to form a closeness category (yes in case of family, (boy)friend, or mentor). Patients were also asked about the (estimated) age of the assailant (categorized into 12–17 years or >18 years), and whether the victim had used alcohol prior to the rape (yes/no).

#### Post-rape characteristics

Patients were asked when they first talked about the rape. The response was used to calculate the disclosure time and the help-seeking time. At the end of the interview, patients were asked whether they had reported to the police after the incident (yes/no), and whether they had received any medical care after the incident (yes/no).

The study was performed in accordance with the precepts and regulations for research as stated in the Declaration of Helsinki, and the Dutch Medical Research involving Humans Subjects Act concerning scientific research. According to the Ethical Medical Committee of the University Medical Centre Utrecht, this act was not applicable to the present study. Written informed consent was obtained from both patients and parents.

### Measures

#### Posttraumatic stress

The Children's Responses to Trauma Inventory (CRTI; Alisic, Eland, & Kleber, 2006) was used for participants aged 12–18 years. This is a 34-item questionnaire assessing severity of PTSD symptoms according to DSM-IV. Patients are asked to indicate to what extent a reaction to a traumatic event was present during the past week. Scores range from 1 to 5, with higher scores indicating more symptomatology. The four subscales: Intrusion, Avoidance, Arousal, and Other Child-Specific Reactions consist of 7, 11, 6, and 10 items, respectively. The reliability of this instrument is good to excellent (Cronbach's α 0.92 for total score, 0.79 for Intrusion, 0.77 for Avoidance, 0.71 for Arousal; Alisic & Kleber, 2010). For the purpose of the study, only the total score was analysed.

#### Depression

Children Depression Inventory (CDI; Kovacs, 1992; Timbremont & Braet, 2002) was used for participants aged 12–17 years of age. The CDI is a 27-item questionnaire, assessing cognitive, affective, and behavioural symptoms of depression. The Dutch CDI has a satisfactory internal consistency, with Cronbach's α ranging between 0.71 and 0.89 (Timbremont & Braet, 2002).

#### Behavioural problems

The Youth Self-Report (YSR; Achenbach & Rescorla, 2001) was used for participants aged 12–18 years. This questionnaire evaluates the teenager's perception of behavioural and emotional problems. YSR has shown to be internally reliable (Cronbach's α's ranging from 0.71 to 0.95), and convergent and discriminant validity is reported to be satisfactory (Bérubé & Achenbach, 2006). The YSR includes four broadband scales and nine narrow-band scales to assess behaviour problems. For the purpose of the study, only the total score on behaviour problems was included in the analyses.

#### General psychopathology

The Symptom Checklist-90-R (SCL-90-R; Arrindell & Ettema, 1986) was used for participants aged 12–25 years. This is a 90-item self-report inventory to assess psychosocial distress. Patients were instructed to indicate the amount they were bothered by each of the distress symptoms during the preceding week. Patients rated 90 distress symptoms on a five-point Likert scale with 1 being “not at all” and 5 being “extremely.” The statements are assigned to eight dimensions, reflecting various types of psychopathology: anxiety, agoraphobia, depression, somatization, insufficiency, sensitivity, hostility, and insomnia. The Global Severity Index (GSI) can be used as a summary of the test and reflects the severity of all answered statements as a global measure of distress. Cronbach's α has been found to range from 0.73 to 0.97. For the purpose of the study, only the GSI was analysed.

### Data analyses

To compare demographic and post-rape characteristics between the early and delayed disclosers, chi-square tests were used. To compare multiple continuous psychological scores, MANCOVA was used with “time since trauma” as a covariate to correct for the potential influence of time since trauma.

Delayed disclosure was used as a dependent variable. The strength of the univariate associations between each potential risk factor and delayed disclosure was estimated by calculating the odds ratio (OR) along with 95% confidence intervals (95% CI). To determine the strongest risk factors for delayed disclosure, each potential risk factor identified in the univariate analyses with a significant OR (*p*<0.05) was entered as a predictor variable into the multivariable model, using a stepwise forward logistic regression (LR) analysis with delayed disclosure as the outcome variable. The Hosmer–Lemeshow goodness-of-fit chi-square was used to calculate how well the data fit the model. For all statistical analyses, a *p*-value of <0.05 was considered statistically significant.

All statistical analyses were conducted using SPSS (IBM SPSS Statistics for Windows, Version 20.0, IBM Corp., Armonk, NY).

## Results

### Socio-demographic characteristics

Socio-demographic characteristics of the sample are presented in Table 1. Victims’ age ranged from 12 to 25 years, with a mean age of 16.7 years (SD=2.7) and a median age of 16.1 years. Victims’ mean age at time of rape was 14.3 years (SD=2.7) and a median age of 13.9 years. Penetration occurred in 79.6% of the cases. None of the victims reported prior chronic child sexual abuse. Data about victim–assailant relationship are presented in Table 2. Victims first disclosed after a mean 20.8 weeks (SD=56.8, range 1–624 weeks), although 58.5% of the cases told within 1 week. First disclosure was to a friend (45.8%), parent(s) (17.1%), (ex) boy-friend (9.4%), family member (6.8%), professional (5.8%), or other adult (15.2%). With regard to post-rape services, 53.8% of all victims consulted a doctor for medical care and 51.4% reported to the police. On average, victims were admitted to the centre 59.8 weeks post-rape (SD=93.7, range 1–676). The mean GSI of the rape victims on the SCL-90-R (*M*=209.7, SD=61.8) was comparable with previously reported data of psychiatric populations [*M=*203.55, SD=61.60; *t*(269)=1.629, *p*=0.104] and was substantially higher [*t*(269)=24.297, *p*<0.001] compared to the general population (*M*=118.28, SD=32.38; Arrindell & Ettema, 1986). For the CDI, mean scores were in the clinical range (*M*=17.2, SD=4.6) and rape victims had significantly higher mean scores (*t*(230)=15,923, *p*<0.001), in comparison to previously reported data of the general population of adolescent girls (Timbremont, Braet, & Roelofs, 2008; *M*=9.01, SD=6.45).

**Table 1:** Demographic characteristics of rape victims (*N*=323) in valid percentages

	*N*	%
Dutch origin^a^	274	84.8
Education level^b^		
Low	182	58.0
Medium	76	24.2
High	56	17.8
Parents divorced	102	31.9
Lives at parental home	273	85.3
Current relationship	81	26.5
Prior negative sex	46	14.8

a Dutch origin was defined as being a child from parents born in the Netherlands

b after 6 years of general primary school, at the age of 12 years, students enter low (4 years), medium (5 years), or high (6 years) secondary education level.

**Table 2:** Victim–assailant relationship (*N*=323) in valid percentages

	*N*	%
Stranger	94	29.5
(Ex-)Boyfriend	32	10.0
Friend	33	10.3
Acquaintance	61	19.1
Person met during nightlife	30	9.4
Second-degree relative	15	4.7
Person seen only once	15	4.7
Person from school	14	4.4
Person met on the internet	12	3.8
Colleague	10	3.1
Mentor	3	1.0

### Differences between early and delayed disclosers

Fifty-nine percent of the sample consisted of early disclosers (disclosure within 1 week). No significant differences in demographic characteristics were found between early and delayed disclosers, except that there were more delayed disclosers in the age category 12–17 years compared to the early disclosers group (*χ* ^2^ (1)=6.96; *p*=0.008). For rape characteristics, significant differences between groups were found for the use of penetration, with more victims of penetration in the delayed disclosers group compared to the early disclosers group (*χ* ^2^ (1)=5.37; *p*=0.02). Also, the delayed disclosers group presented more victims of verbal and/or weapon threats than the early disclosers group (*χ* ^2^ (1)=5.35; *p*=0.02). Furthermore, among the delayed disclosers more victims identified the assailant as a close person compared to the early disclosers (*χ* ^2^ (1)=10.84; *p*=0.001). Alcohol was used more often in the early disclosers group compared to the delayed disclosers group (*χ* ^2^ (1)=20.24; *p*<0.001).

With respect to post-rape characteristics, a significantly smaller proportion of the delayed disclosers (15.9%) utilized medical services following the rape compared to the early disclosers (30.3%; *χ* ^2^ (1)=5.32; *p*=0.02). Similarly, a significantly smaller proportion of the delayed disclosers (14.6%) compared to the early disclosers (34.3%) reported the rape to the police (*χ* ^2^ (1)=16.15; *p*<0.001). The time since trauma at admission was significantly lower for early disclosers (*M*=41.1 weeks, SD=79.4) than for delayed disclosers (*M*=82.9 weeks, SD=103.3*; t*(314)=4.06, *p*<0.001). Mean and median time to seek help were 37.7 and 12.0 weeks, respectively. Mean time to seek help did not differ between groups (* t*(309)=2.54, *p*<0.48). Excluding outliers (*M*±3 SD, *N*=11) did not change the outcome of this analysis. Both early and delayed disclosers scored in the highest level of psychological distress when compared to previously reported norm scores (CRTI, Alisic, Eland, Huijbregts, & Kleber, 2012; CDI, Timbremont et al., 2008; YSR, Achenbach & Rescorla, 2001; SCL-90, Arrindell & Ettema, 1986), but the MANCOVA results showed that when comparing multiple continuous psychological scores, the overall psychological functioning (posttraumatic stress, depression, behavioural problems, and general psychopathology) did not differ significantly between early and delayed disclosers (*F*(6,198)=0.88, *p*=0.51).

Table 3 shows the ORs with 95% CIs for the associations between potential risk factors and delayed disclosure. Delayed disclosers, when compared to early disclosers, were significantly more likely to be in the age category of 12–17 years (OR=2.10), to have experienced rape by a close person (OR=2.35), to have been threatened verbally and/or with a weapon (OR=1.75), and to have experienced penetration (OR=1.99). Delayed disclosers were also found less likely to have used alcohol prior to the rape (OR=0.22). None of the other factors were found to be significant risk factors for delayed disclosure.

**Table 3:** Demographic and (post-)rape characteristics by disclosure time (early vs. delayed disclosers) and odds ratios for delayed disclosure

	Early disclosure (*N*=185)		Delayed disclosure (i.e., >1-week post-rape), *N*=131			
			
Demographic and (post-)rape characteristics	*N*	%	*N*	%	OR	95% CI
Age category (years)						
18–25	55	17.4	22	7.0		
12–17	130	41.1	109	34.5	2.10	1.20–3.65*
Dutch origin						
No	27	8.5	22	7.0		
Yes	158	50.0	109	34.5	0.85	0.46–1.56
Living with parent(s)						
No	29	9.2	16	5.1		
Yes	155	49.2	115	36.5	1.35	0.70–2.59
Complete family structure						
No	58	18.4	42	13.3		
Yes	127	40.3	88	27.9	0.96	0.59–1.55
Current sexual relationship						
No	127	41.8	97	31.9		
Yes	53	17.4	27	8.9	0.67	0.39–1.14
Prior negative sexual experience(s)						
No	152	49.4	110	35.7		
Yes	32	10.4	14	4.5	0.61	0.31–1.19
Known assailant						
No	56	17.7	36	11.4		
Yes	129	40.8	95	30.1	1.15	0.70–1.88
Close to assailant						
No	150	47.6	84	26.7		
Yes	35	11.1	46	14.6	2.35	1.40–3.93*
Group rape						
No	160	50.8	116	36.8		
Yes	24	7.6	15	4.8	0.86	0.43–1.71
Age of assailant (years)						
12–17	63	20.6	54	17.6		
>18	117	38.2	72	23.5	0.72	0.45–1.14
Use of penetration						
No	46	14.7	19	6.1		
Yes	136	43.5	112	35.8	1.99	1.10–3.60*
Use of threats						
No	90	31.6	48	16.8		
Yes	76	26.7	71	24.9	1.75	1.09–2.82*
Use of physical violence						
No	130	42.6	82	26.9		
Yes	51	16.7	42	13.8	1.31	0.80–2.14
Victim's alcohol use						
No	72	33.5	69	32.1		
Yes	61	28.4	13	6.0	0.22	0.11–0.44*

* *p<*0.05.

Seven participants were dropped from analyses due to missing disclosure time data.

### Predicting delayed disclosure

A stepwise forward LR analysis was conducted to predict delayed disclosure, using “age category,” “close assailant,” “use of threats,” and “penetration” as predictors. Victims’ alcohol use was not entered in the analysis because of missing values for 33.4% of the cases. The use of threats was not a significant predictor in the model. A test of the full model against a constant-only model was statistically significant, indicating that the predictors (i.e., age category 12–17 years, close assailant, penetration) reliably distinguished between early and delayed disclosers (*χ* ^2^ (3)=23.09, *p*<0.000). There were no significant interactions between the predictors. Nagelkerke's *R* ^2^ of 10.5% suggests only a modest association between the predictors and delayed disclosure, although the model did show an adequate fit to the data (Hosmer–Lemeshow *χ* ^2^ (4)=2.77, *p*<0.60). In total, 62% of the respondents were categorized correctly, when using the three predictors that contributed significantly to the prediction of delayed disclosure: age category 12–17 years (OR 2.05, CI 1.13–3.73), penetration (OR 2.36, CI 1.25–4.46), and closeness to the assailant (OR 2.64, CI 1.52–4.60).

## Discussion

The results of this study show that, although no differences were found between delayed and early disclosers in psychological functioning and time to seek help, delayed disclosers were less likely to use medical services and to report to the police than early disclosers. Furthermore, this study identified a number of factors related to the timing of rape disclosure, showing that delayed disclosers represented significantly more adolescents than young adults, significantly more victims of penetration than assault, significantly more victims who were threatened than not threatened, and significantly more victims who were close with the assailant.

The finding that delayed disclosers are less likely to utilize medical services and report to the police than early disclosers is in line with previous studies in adult women (Ahrens et al., 2010; Ullman, 1996; Ullman & Filipas, 2001). It suggests that disclosure latency is important for public health and safety, as delayed disclosure may not only impede reception of proper medical care, such as treating anogenital injuries and preventing the onset of STDs and unwanted pregnancy (Linden, 2011), but also impede the forensic investigation and apprehension of the assailant (Lacy & Stark, 2013).

Three variables were identified that successfully predicted delayed disclosure: age category 12–17 years, penetration, and the assailant being a close person. The finding that the victim's age significantly predicts disclosure latency is in line with previous research showing that adolescents are at a greater risk for delayed disclosure when compared to their older counterparts (Kogan, 2004; Smith et al., 2000). Adolescents may be less able to overcome the barriers to disclose, including factors such as assailant tactics for maintaining secrecy, stigma that often accompanies rape, and fear that their parents would consequently limit their freedom (Crisma, Bascelli, Paci, & Romito, 2004). Also, as victims approach adulthood, they may possess more information about their rights and options after victimization, and have more possibilities for whom to disclose. In our study, most adolescents disclosed the rape event to peers, in line with prior research (Crisma et al., 2004; Priebe & Svedin, 2008).

The use of penetration was found to make victims more likely to postpone disclosure, opposite to the results from Priebe and Svedin (2008), but in line with an older study by Arata (1998), who found that more severe forms of sexual abuse were associated with less disclosure. Penetration may influence disclosure latency through a variety of mechanisms. It could be argued that more severe rape, indicated by the use of penetration, is more likely to be accompanied by extensive coercive use of tactics to maintain the victim's silence, with fear of reprisal possibly contributing to the finding of delayed disclosure (Kogan, 2004). Also, adolescents may think that social reactions in response to disclosure are more negative in case of completed rape compared to assault.

Another factor that seems to make immediate disclosure of rape less likely is closeness to the assailant, as indicated by the assailant being a (boy)friend, family member, or mentor. This finding is consistent with previous studies showing that the closer the relationship between the victim and assailant, the less likely the young woman was to report this victimization to anyone (Koss, 1988; Rickert et al., 2005; Wolitzky-Taylor et al., 2011). The dynamics of intrafamilial abuse is often proposed as the explanation for delayed or non-disclosure (Kogan, 2004; Smith et al., 2000). In the present study, however, only 5% of the assailants were identified as a family member. Most close relationships referred to (boy)friends, suggesting that a significant percentage of the sample experienced peer-to-peer victimization. This type of victimization is most likely to occur during adolescence, as compared to childhood or young adulthood, and greatly increases the risk of revictimization (Humphrey & White, 2000). Hence, victims of rape by peers may be a target group for interventions promoting early disclosure.

Clearly, there are many variables working in tandem to affect the timing of victim's disclosure. A closer look at the final model, which identified three unique variables that contributed significantly to the prediction of delayed disclosure, can help us to better understand the phenomenon of initial disclosure in adolescents and young adults. Younger adolescent victims who are raped by a close person are more likely to delay disclosure than older victims of attempted rape by a stranger or acquaintance. Perhaps, they struggle with the notion that someone close to them performed such a violent act against them, which confuses them about what might happen in terms of safety if they would disclose (or not). This finding is especially important in the light of the fact that approximately 80% of victims had some sort of relationship with their perpetrator prior to the assault (Basile, Chen, Black, & Saltzman, 2007). With regard to rape types, it would intuitively seem that less severe forms of sexual assault are associated with delayed disclosure and that completed rape would be easier to identify as clearly inappropriate and wrong. Victims of completed rape, however, may be more likely to experience negative psychological reactions, e.g., self-blame and avoidance coping. It is conceivable that they delay their disclosure as a result of rape-induced psychological distress (Starzynski, Ullman, Filipas, & Townsend, 2005), not necessarily the severity of the assault.

Although the final model showed acceptable goodness of fit, the percentage of explained variance of delayed disclosure was modest. Thus, there must be other variables predictive of delayed disclosure, such as the assailant's use of alcohol or weaker support systems, that we did not assess in this study. Besides this limitation, there are other drawbacks of this study that should be mentioned. First, a clinical sample was used with patients reporting high mean levels of psychological distress. This ceiling effect may explain why no differences were found between early and delayed disclosers on psychological functioning, contrary to prior studies (Broman-Fulks et al., 2007; Ruggiero et al., 2004). Second, posttraumatic stress was only assessed for children up to 18 years, and for young adults additional suitable measures were not used. Third, information could have been lost due to dichotomizing the variable disclosure latency. Fourth, results may not be generalizable to all rape victims, because the percentage of victims that consulted a medical professional and reported to the police was higher in our sample than in most studies (Hanson et al., 2003; Resnick et al., 2000; Zinzow, Resnick, Barr, Danielson, & Kilpatrick, 2012). Perhaps, these differences could, at least partially, be explained by the fact that stranger rape, representing 30% of our sample, leads to higher likelihood of help-seeking and police reporting because of its association with higher acknowledgment of victim status (Resnick et al., 2000; Smith et al., 2000). The fact that this is a help-seeking sample is critical for the reasons cited in the discussion, but also because the generalizability of these data to rape victims who never tell anyone—perhaps the group most at risk—simply cannot be known. Besides these limitations, several strengths of the current study need to be noted. One strength is the unique set of adolescents and young adults who presented at a mental health care centre after a single rape event, but who reported no prior chronic sexual abuse in childhood. For 85% of the sample, the index trauma was a first time rape. Moreover, data were collected at a designated referral centre for victims of rape and, therefore, the sample is likely to represent the clinical population of Dutch victims in the age group of 12–25 years.

The findings of the current study, suggesting that delayed disclosers are less able to benefit from emergency medical care and evidence collection, have a number of practical implications. One of the strategies to enhance victims’ willingness to disclose within the first week post-rape may be *sexual* education campaigns in school and media, as being uninformed is one of the reasons for them not to disclose (Crisma et al., 2004). Education may include medical information on rape-related pregnancy and STDs, as well as the need for timely emergency contraception and prophylaxis, given that these concerns appear to be facilitators of seeking medical help (Zinzow et al., 2012). Also, practical information about DNA evidence and how to best protect it, e.g., related to showering, clothing, eating, and drinking, may increase the awareness of opportunities in the early-phase post-rape. Moreover, facts about the potential psychological impact of rape, such as PTSD and revictimization, but also information about evidence-based treatments (Elwood et al., 2011; Littleton & Ullman, 2013; McLaughlin et al., 2013), may increase help-seeking behaviour in an early stage. Furthermore, efforts to encourage early disclosure must consider peer-to-peer victimization as a primary factor, as most participants in this study experienced this type of victimization, and may initially not have defined *or acknowledged* the incident as rape because they rationalize such experiences as normal (Hlavka, 2014), leading to the finding of delayed disclosure.

In conclusion, the results of the present study suggest that adolescent victims of rape with penetration by someone close are at increased risk for delayed disclosure, and that delayed disclosers are less likely to use medical services and to report to the police. These findings may assist clinicians and policymakers in understanding rape and help to develop interventions (Unterhitzenberger & Rosner, 2014), specifically targeted to support adolescents and young adults to disclose in an early-phase post-rape. Although the vast majority of the participants was living at their parental home, many of the sample did not first disclose to their parents. Therefore, it could be argued that in prevention programs specific attention should be given to the strengthening of the child–parent relationship, to facilitate disclosure to parents (Schönbucher et al., 2012). Next, as victims tend to disclose mostly to peers, prevention programmes may need to aim at teaching adolescents how they can help a peer victim if they become a recipient of disclosure (Schönbucher et al., 2012). In addition, education may increase victims’ willingness to disclose early, thereby increasing opportunities for access to health and police services. It is more likely to reach adolescents with direct, active, and online outreach programs via communication channels that are frequently used by adolescents and young adults particularly social media (i.e., Facebook, Twitter, YouTube, etc.), forums, and mobile apps. Such programmes, wherein adolescents and young adults are being treated as agents and decision makers (Hlavka, 2014), should focus on information concerning what rape actually is—not only the stereotypical idea of rape and what (not) to do in the aftermath of rape especially in the first week post-rape. Another way to help improve the support of victims of rape is the implementation of multidisciplinary sexual assault centres (Bicanic, Snetselaar, De Jongh, & Van de Putte, 2014; Bramsen, Elklit, & Nielsen, 2009), as these may be the most suitable places to organize education campaigns and offer integrated post-rape services in one location. Future research should investigate whether the availability of such centres increases the prevalence of police reporting and use of medical care. Moreover, as discussed, previous research concerning the topic of disclosure has focused on the disclosure process, mainly the effect of negative social reactions, and not the latency. In future research, social reactions in relation to disclosure (latency) should be assessed by using the Social Reactions Questionnaire, as well as the victim's perception of their own experience being defined as rape, as many girls and young women do not report or seek help because they regard sexual violence against them as normal (Hlavka, 2014).
